# Lumbosacral fusion increases the risk of hip osteoarthritis

**DOI:** 10.1186/s13018-023-03932-0

**Published:** 2023-06-24

**Authors:** İbrahim Ulusoy, Aybars Kıvrak

**Affiliations:** 1 Department of Orthopedics and Traumatology, Diyarbakır Selahaddin Eyyubi State Hospital, Diyarbakir, Turkey; 2 Department of Orthopedics and Traumatology, Avrupa Hospital, Adana, Turkey

**Keywords:** Lumbosacral fusion, Hip joint, Adjacent segment, Spinopelvic, Joint space narrowing

## Abstract

**Objective:**

There may be biomechanical changes in the adjacent hip joint after lumbosacral fusion. The literature has limited information on how these biomechanical changes may result in hip joint space.

**Material:**

**method:**

Our retrospective study examined hip joint space narrowing in patients who underwent lumbosacral fusion between 2020 and 2022. In addition, spinopelvic parameters such as sacral slope, the sagittal vertical axis, pelvic incidence, lumbar lordosis, and pelvic tilt were compared in patients who underwent short-segment (up to three levels, S group) and long-segment (4 and higher levels, L group) fusions.

**Results:**

Our study found no significant relationship between spinopelvic parameters and joint space narrowing in the S and L groups. In addition, it was determined that there was more narrowing in the hip joint space in the long-segment group, and there was a positive correlation between the segment length and the narrowing in the hip joint space.

**Conclusion:**

After lumbosacral fusion, narrowing of the hip joint space was observed. Particularly patients with long-segment lumbosacral fusion should be followed closely regarding hip osteoarthritis risk.

## Introduction

Spinal fusions can be performed for many reasons, such as degenerative scoliosis, trauma, spondylolisthesis, spinal deformities, and radiculopathy (due to foraminal stenosis). When done with the right indication, this treatment method improves the patients’ daily living activities. However, spinopelvic biomechanical changes may occur in this operation.

Adjacent segment degeneration is an undesirable condition seen after spinal fusion and is not uncommon [1]. After lumbosacral fusion, changes in the forces acting on the sacrum and pelvis may occur. In addition, the mobility of the acetabulum may be inhibited, and the risk of impingement increases [2]. Due to such reasons, there needs to be more literature about what kind of consequences can occur in the hip joint after lumbosacral fusion and when it occurs.

There are limited publications in the literature on the effect of lumbosacral fusions on the hip joint. There is no publication in the literature on the effect of isolated lumbosacral fusions on the hip joint. Our study measured the effects of spinopelvic measurement parameters and fusion segment length on the change in hip joint space distance in patients who underwent lumbosacral fusion.

## Material method

Clinical research ethics committee approval was obtained for this study. The ethics committee number is 117–1742. Our retrospective study examined patient files between 2020 and 2022 in a single center. Patients who underwent lumbosacral fusion (operated for scoliosis, trauma, spinal deformities, and foraminal stenosis) were included in the study. Those who had previous hip surgery, missing or conflicting information in their files, finding or history of hip arthrosis before spinal surgery (e.g., Kellgren–Lawrence grade ≥ II) [3], no preoperative hip radiographs or not having hip radiographs taken appropriately, who underwent spinal fusion revision, had lower extremity height difference, had rheumatic diseases, had spinal cord injury, were under the age of 18, and had a follow-up period of less than one year were excluded from the study. Patients who underwent lumbosacral fusion were divided into short-segment (up to three levels, S group) and long-segment (4 and higher levels, L group) spinal fusion patients. Demographic data of the patients were recorded.

### Radiographic evaluation

Operated patients have whole-spine standing 2-way radiography taken before routine surgery. Early postoperative radiographs were also taken routinely for these patients. Spinopelvic alignment parameters of the patients were obtained by making measurements on the radiographs taken during the postoperative 3rd month. Sacral slope (SS), sagittal vertical axis (SVA), pelvic incidence (PI), lumbar lordosis (LL), thoracic kyphosis (TK), and pelvic tilt (PT) were measured as described in previous studies [4–7]. Radiological measurements were made with the help of Extreme PACS (Turkey). Program measurement precision is 0.1 mm. As the minimum joint width (MJW), the narrowest point from the lateral edge of the acetabulum and fovea was determined. MJW of the hip was measured from the same point preoperatively and postoperatively. Postoperative MJW measurement was obtained with the help of X-rays taken at the last follow-up. The narrowing of the joint distance was calculated according to the follow-up time with the formula previously described in the literature (formula: [preoperative MJW (mm) − postoperative MJW (mm)]/follow-up years) [8]. Two orthopedic specialists made radiographic measurements. The same surgeons repeated the measurements with an interval of two weeks, and the final values were found by taking the averages.

### Statistics

Statistical analyses were performed using the Statistical Package of the Social Sciences (IBM SPSS 28.0.1.0; Corp., Armonk, NY, USA). The variables were investigated using visual (histograms, probability plots) and analytical methods (Kolmogorov–Smirnov/Shapiro–Wilk’s test) to determine whether or not they are normally distributed. The correlation coefficients and their significance were calculated using the Pearson test. Multiple regression analyses were performed to identify the independent predictors of the rate of joint space narrowing. These tests were chosen within the framework of the general rules in statistics, depending on the characteristics of the dependent and independent variables. The significance level was set at *p* < 0.05.

## Results

One hundred and thirteen patients who met the inclusion criteria were reached. Considering the exclusion criteria, the data of 101 patients were reached. Two hundred and two hips were evaluated. Short-segment fusion was performed in 55 of these patients, and long-segment fusion was performed in 46. The patient’s demographic data, the duration of follow-up, and the average of the radiological measurement values are given in Table 1, and the correlation between the MJW and variables obtained by considering the follow-up period as described in the literature is given in Table 2. A positive correlation was found between the number of segments fused and MJW. The results of linear multiple regression analysis are given in Table 3. Multiple regression analysis revealed the number of fusion segments as independent risk factors for joint space narrowing.

**Table 1:** Demographic data of patients, follow-up times, and average radiological measurement values

	S group (n^p^ = 55, n^h^ = 110)	L group (n^p^ = 46, n^h^ = 92)	Total (n^p^ = 101, n^h^ = 202)
Age	59.79 ± 12.91	57.22 ± 13.56	58.62 ± 13.2
Gender (male/female)	32/23	23/23	55/46
Number of segments	2.83 ± 0.37	5.02 ± 1.84	3.83 ± 1.67
Follow-up time (months)	23.92 ± 8.04	22.91 ± 7.1	23.46 ± 7.6
PI (degrees)	47.67 ± 9.44	52.46 ± 10.33	49.85 ± 10.09
PT (degrees)	22.01 ± 7.86	26.84 ± 8.04	24.21 ± 8.27
SS (degrees)	25.45 ± 9.18	27.99 ± 6.28	26.14 ± 7.52
SVA (mm)	42.34 ± 22.14	55.44 ± 29.86	48.31 ± 26.63
LL (degrees)	36.12 ± 18.09	26.95 ± 15.82	31.85 ± 17.78
MJW*	0.06 ± 0.05	0.09 ± 0.07	0.08 ± 0.06
TK (degrees)	27.26 ± 3.86	25.83 ± 4.09	26.61 ± 4.01

*Value calculated according to the formula [5]. n^p^: Number of patients; n^h^: Number of hips

**Table 2:** Correlation between MJW* and variables

		MJW*	
	*r*	*P* Value	*N*
Age	0.01	*0.919*	101
Gender(male/female)	0.28	*0.782*	101
Number of segments	0.671	** < *0.001***	101
Follow-up time (months)	0.16	*0.871*	101
SS (degrees)	0.002	*0.985*	101
SVA (mm)	− 0.010	*0.924*	101
PI (degrees)	− 0.095	*0.347*	101
LL (degrees)	− 0.078	*0.438*	101
PT (degrees)	0.013	*0.896*	101
TK (degrees)	− 0.125	*0.213*	101

*p* < 0.05 is significant

*MJW value calculated according to the formula [8]. *N*: number; *r*: The correlation coefficient

**Table 3:** Results of linear multiple regression analyses

	Unstandardized coefficients		Standardized coefficients	t	Sig
	B	Standard error	Beta		
Age	0.000043	0.000367	0.009	0.117	*0.907*
Number of segments	0.035	0.004	0.898	9.191	< *0.001*
Follow-up time (months)	− 0.000309	0.001	− 0.036	− 0.475	*0.636*
SS (degrees)	− 0.000116	0.001	− 0.014	− 0.188	*0.852*
SVA (mm)	− 0.00008	0.000193	− 0.033	− 0.414	*0.680*
PI (degrees)	− 0.000184	0.00047	− 0.03	− 0.391	*0.697*
LL (degrees)	− 0.000245	0.000291	− 0.066	− 0.842	*0.402*
PT (degrees)	0.000233	0.001	0.03	0.373	*0.71*

*p* < 0.05 is significant

According to the ICC estimates, values less than 0.5 are poor, values between 0.5 and 0.75 are moderate, values between 0.75 and 0.9 are good, and values greater than 0.90 indicate excellent reliability [9]. Inter- and intra-reader reliabilities were excellent in the measurements made.

## Discussion

Our study observed that there was more narrowing of the hip joint space in the long-segment group in patients who underwent lumbosacral fusion. In addition, a positive correlation was found between segment length and joint space narrowing. It has been revealed that the narrowing of the hip joint space is greater as the segment length increases.

Spinal fusion is a surgical treatment method with promising results in selected cases. In addition to these promising results, there may be some undesirable results. Degeneration of the adjacent segment due to biomechanical reasons after fusion is one of them [10]. The frequency of adjacent segment disease (ASD), which can be counted as a mid- and long-term complication, ranges from 5.2 to 49% [1]. We can consider the hip joint as an adjacent segment in lumbosacral fusions. In addition, spinopelvic mobility may change after lumbosacral fusion, and changes may occur in the mechanical loads on the hip joint. In addition, there is a risk of impingement in the hip due to decreased mobility of the acetabulum [2].

As one of the spinopelvic parameters and the sum of SS and PT, changes in pelvic incidence affect the spine’s biomechanics and sagittal balance. In some studies, it has been revealed that there is a significant correlation between PI and hip osteoarthritis [11, 12]. In addition, in the study of Kawai et al. [7], the high PI value was associated with hip osteoarthritis. The risk of developing hip osteoarthritis is thought to be higher in patients with high PI in lumbosacral fusions due to a higher load on the pelvis [7]. Some publications argue that there is a positive correlation between SS values and PT values and the development of hip osteoarthritis [6, 13, 14]. In this case, as in patients with high PI, excessive load on the hip joint is blamed. In addition, a study by Kumaran et al. [15] found that the stress to which the hip is exposed during many movements increases in patients with high SS degrees.

Contrary to these publications, no relationship was found between PI value and hip osteoarthritis in a study [16]. Although many studies reflect the relationship between PI and hip osteoarthritis, there is no consensus in the literature [17]. In addition, in the meta-analysis of Wang and Ding, no relationship was found between SS and PT values and adjacent segment disease [1]. Our study found no relationship between SS, PT, and PI values and the change in distance in the hip joint space. The sagittal vertical axis is important in determining the center of gravity. In addition, it is an important parameter when planning spinal deformities. A positive correlation was found between SVA and coxarthrosis in the publications. In the study of Miyagi et al., it was observed that the increase in SVA caused the progression of coxarthrosis [18]. Another publication concluded that the load on the hip joint increased with the increase in SVA [19]. There are many publications in the literature examining the relationship between ASD and LL (which is another sagittal spinal parameter). In the study of Djurasovic et al., it was concluded that the loss of LL results in ASD [20]. In work written by Nakashima et al., it was concluded that providing appropriate LL plays an important role in preventing the development of ASD [21]. It has been concluded that ASD occurs due to non-physiological load transfer to the adjacent segment after hypolordosis [21]. Our study found no relationship between SVA and LL values and the change in distance in the hip joint space.

The physiological movement of the body changes somewhat after spinal fusion. If we consider the musculoskeletal system as a whole, as a result of the restriction of spinal movement, an imbalance occurs in the loads on the hip joint [8]. A mobile spinal joint can compensate for the load on the hip, but this compensation mechanism does not work because the movement decreases in long fusions. We attribute more narrowing of the hip joint space to this as the fusion level gets longer.

There are some limitations of our study. The first conspicuous limitation is the retrospective nature of our study. In addition, our mean follow-up period is 23.46 months. Evaluation of long-term follow-up results is needed to obtain more detailed information. In addition, the fact that the cartilage thickness and joint fluid could not be evaluated can be considered a limitation.

## Conclusion

The interrelationship between spinopelvic motion and its impact on the hip joint is a complex biomechanical and dynamic phenomenon. The axial skeleton should be evaluated as a whole in cases requiring spinal fusion. In addition, it should be considered that as the number of segments fused increases, the probability of narrowing of the hip joint will be greater. Patients should be followed closely regarding hip joint osteoarthritis, and modifiable risk factors such as lifestyle modification should be focused on.

## Data Availability

The datasets used and analyzed during the current study are available from the corresponding author on reasonable request.
